# Implementation of Key Components of Evidence-Based Family Therapy for Eating Disorders in Child and Adolescent Psychiatric Outpatient Care

**DOI:** 10.3389/fpsyt.2020.00059

**Published:** 2020-02-20

**Authors:** Ulf Wallin, Sanjib Saha

**Affiliations:** ^1^ Centre of Eating Disorders, Psychiatry Skåne, Lund, Sweden; ^2^ Health Economics Unit, Department of Clinical Science (Malmo), Lund University, Lund, Sweden

**Keywords:** eating disorders, anorexia nervosa, children, adolescents, family based treatment, economic evaluation

## Abstract

**Background:**

Restrictive eating disorders with pronounced starvation are serious psychiatric conditions that often begin during childhood or adolescence. An early and efficient intervention is crucial to minimize the risk of the illness becoming longstanding and to limit the consequences. There is good evidence that weight gain during the first month of treatment provides a better prognosis. Only a limited amount of young people suffering from severe restrictive eating disorder receive an evidence-based treatment at present in Sweden. The ROCKETLAUNCH project intends to implement key components of the evidence-based family therapy during the first month of treatment in child and adolescent psychiatric outpatient care.

**Methods:**

From the southern part of Sweden, 12 local child and adolescent psychiatric outpatient services will take part. All patients with a restrictive eating disorder and pronounced starvation together with their families will be asked to take part in the study. We expect that one hundred 50 patients will be assessed every year. The patients and their families will receive 1 month of intense manualized treatment. Body weight, days in inpatient care, eating disorder, and other psychopathology-related symptoms, will be evaluated after one month and at 12-month follow-up. Economic evaluation of ROCKETLAUNCH will also be carried out alongside the intervention. At each outpatient clinic, data from the 10 previous patients will be gathered to compare the treatment provided at ROCKETLAUNCH with the standard treatment in Sweden.

**Discussion:**

We expect that by implementing the key components of the evidence-based family therapy during the first month of treatment, the prognosis of young newly diagnosed patients with severe restrictive eating disorders, primarily anorexia nervosa will improve, which, in turn, will reduce the need for psychiatric inpatient care.

**Clinical Trial Registration:**

www.ClinicalTrials.gov, identifier NCT04060433.

## Introduction

Restrictive eating disorders with pronounced starvation is one of the most acute and life-threatening conditions in child and adolescent psychiatry. Severe restrictive eating disorders in young people are primarily anorexia nervosa (AN), atypical anorexia nervosa (AAN), and avoidant/restrictive food intake disorder (ARFID). It is crucial to end the starvation with treatment and achieve medical stabilization with weight gain for those who are underweight (1). Current research indicates that weight gain during the first month of treatment leads to a better prognosis (2–5). The family-based treatment has the best support in research to achieve weight gain for adolescents with AN (6). Intensive family treatment which focuses on terminating starvation has proven to be able to achieve sustainable results even after 30 months (7). However, only 26.4% of children and adolescents with an eating disorder in Sweden receive any type of family therapy according to RIKSÄT, the national quality register for eating disorders (8). The first treatment that many young patients with an eating disorder receive is inpatient care. According to the RIKSÄT report inpatient care is the first registered treatment for 25% of the patients who are under 18 and with a mean age of 15 years. This underlines the need for a better initial treatment. Prompt use of manualized family therapy in the outpatient setting may prevent hospitalization, as well as improve the prognosis.

In order to improve the treatment of the patients below 18 years of age with a restrictive eating disorder and pronounced starvation within Region Skåne (i.e., the healthcare provider for the province of Scania), a working group was assigned to design guidelines for Child and Adolescent Mental Health Services (CAMHS) in Skåne. The working group consisted of representatives from the five local CAMHS units with eating disorder teams in Skåne, as well as the regional unit at Region Skånes Center of Eating Disorders in Lund. Pathways into services and initial treatment processes and outcomes were examined. Each service examined its 10 most recent patients. Information from 60 patients was collected. The working group found that the time between referral and first visit differed between units (from 12.1 to 23.7 days). The number of visits during the first month was 6.2 visits, on average (from 4.7 to 7.6) just over one visit per week. Some of these visits were in the scope of day-care. The rate of weight gain during the first month varied between the different services from 0.1 to 1.5 kg. It is lower than 2.0 kg, which is the lowest level of weight gain during the first month which international research has shown indicates a better prognosis (5).

Based on the data presented above, it was found that there is a need for service delivery improvement in three main areas:

1. For treatment to begin with less delay upon first referral.
2. Improve treatment by implementing the evidence-based treatment model.
3. Offering equal care, regardless of catchment area.

As part of this development work, the working group devised basic guidelines for intensive treatment during the first month: an evidence-based treatment focused on helping the patient normalize eating and terminate starvation. Treatment with a focus on normalizing the eating will continue even after the first month.

## Aim of the Study

The working group identified some gaps and the need for better treatment. Therefore, we are providing the ROCKETLAUCH intervention in a small scale to see the effectiveness and cost-effectiveness. If successful, there may be a scope for nationwide implementation.

The ROCKETLAUNCH project aims to improve treatment by adding important key components of evidence-based practice to the treatment given today. The project is trying to close the gap between what is known about effective treatment and what is actually provided (9). By focusing on key element in the family therapy model we also hope to make it easier to implement. This has been tested in a primary care setting in a study where it seems to be a feasible way (10).

There are six central areas regarding research and evaluation.

1. Are the prerequisites for implementing the guidelines for ROCKETLAUNCH satisfactory? We want to assess how the clinicians that will carry out the treatment in the project experience opportunities and difficulties.
2. Are the waiting times shortened and is the recommended treatment intensity maintained? An important part of the study is to see if the healthcare system can improve its care for these life-threatening disorders.
3. Will the need for inpatient care be reduced?
4. Will weight gain improve as expected? The number of patients that gained 2 kg or more before the project has been 23.7%. This means that a greater proportion of the intervention group should gain 2 kg or more during the first month of the treatment. In a study of family-based treatment (FBT), 35% of the patients gained 1.8 kg during the first 4 weeks (4), which should be the proportion that the project aims for.
5. Does the family meal make a difference for those patients that do not gain weight? One particular issue is to evaluate the effect of family meals that will only be offered to those patients who do not gain weight as expected. The importance of family meals has been very sparingly evaluated in research (11). In treatment studies, there is no clarity on whether the family meal is needed. There may even be indirect support that the family meal is not crucial to the outcome (12, 13). In one study comparing separated family therapy *versus* conjoint family therapy and another comparing parent-based therapy (PBT) with FBT there were no differences in outcome. In both studies, one treatment arm did not offer a family meal. On the other hand, there may be support for providing an additional family meal when the weight gain is not satisfactory and treatment has to be intensified (14).
6. Is the ROCKETLAUNCH intervention cost-effective comparing to the standard care both at the short-term and long-term duration?

The hypothesis in the study is that by implementing key components of the evidence-based family therapy during the first month of treatment, this will improve the prognosis of young newly diagnosed patients with severe restrictive eating disorders, primarily AN, and reduce the need for psychiatric inpatient care.

## Methods

**Figure 1:**
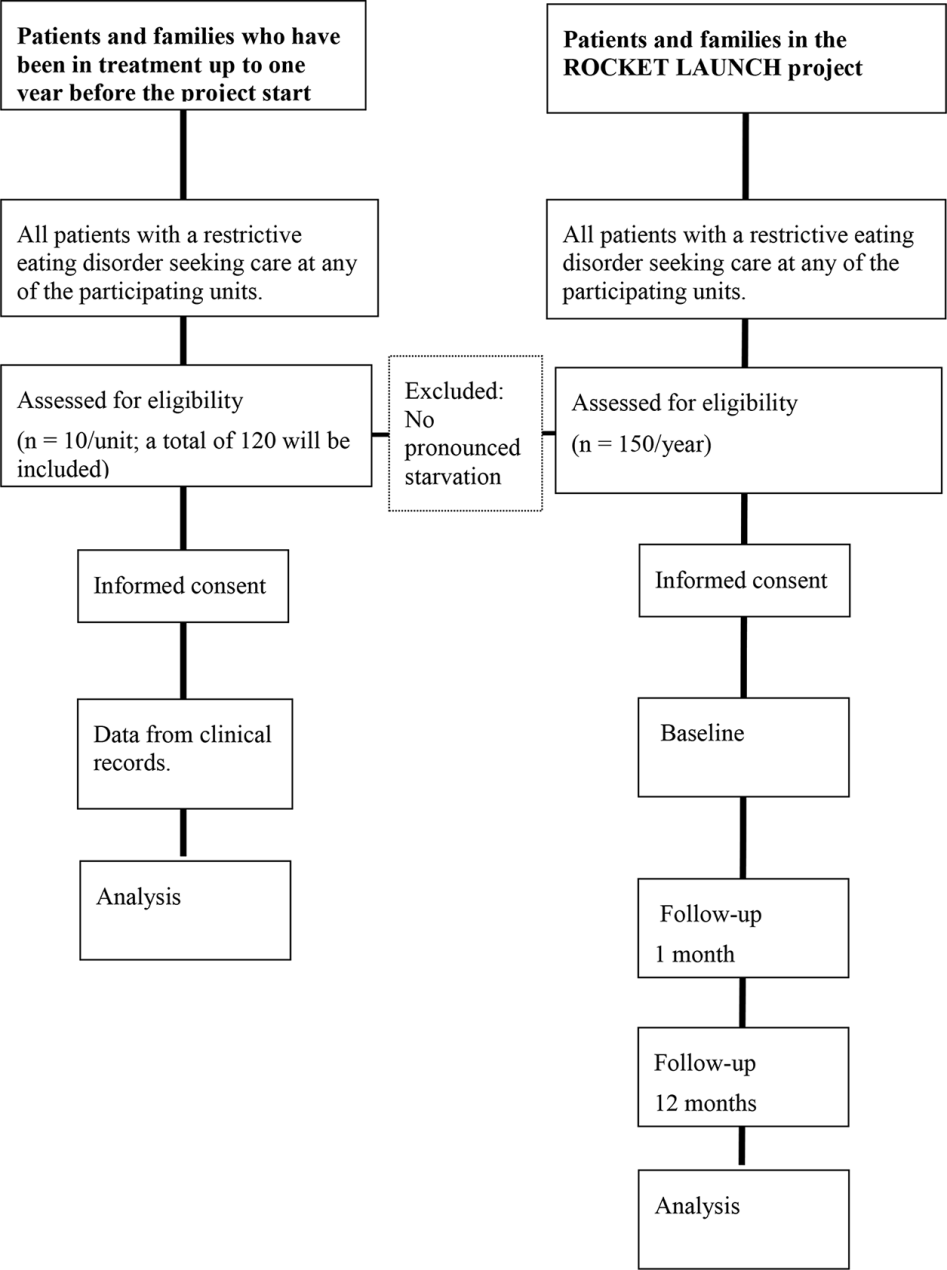
Provisional flowchart.

The study is a single group assignment, with a historical comparison group to evaluate the implementation of evidence-based treatment at 1 month and 12 months follow-up. The provisional flowchart is presented in **Figure 1**.

The total study duration will be 4 years. The participation duration for study subjects will be 12 months, i.e., a 1 month treatment period followed by an evaluation 11 months after the end of the intervention.

### Participants

The project covers the Southern Healthcare Region in Sweden, and its eating disorder units in the counties of Skåne, Halland, and Blekinge will participate in the project. In addition, AnorexiBulimiCenter in Kalmar and Västervik are also involved in the project.

Patients should all be below 18 but over 7 years of age. The inclusion criteria for the patients are that they fulfill one of the three diagnoses: AN, AAN, or ARFID and that they are in pronounced starvation. The inclusion of ARFID with pronounced starvation is justified by the fact that the treatment needs to focus on weight gain and medical stabilization during this first month of treatment. There are not much research yet on which treatment is most effective, but there are indications that key components of FBT might be crucial during the first phase of treatment (15). All patients that fulfill the inclusion criteria and their parents will be asked to participate in the research. Each CAMHS collects information from 10 previously treated patients who meet the same inclusion criteria and have completed the 1 year observation period before the ROCKETLAUNCH project starts. Informed consent is obtained by mail from the previous patients and their parents. All patients with severe restrictive eating disorders and their families in Skåne, Halland, Blekinge, and Kalmar will be offered to participate in the project. A total of 12 out-patient services will provide the treatment.

The exclusion criteria are patients who are diagnosed with psychosis, mental deficiency, organic cerebral disorder, or any pathology that interfere with feeding or cause regurgitation. Patients who does not speak Swedish and patients who are already in any form of family therapy for other conditions will also be excluded.

### Intervention

The general guidelines for the initial treatment of restrictive eating disorders with pronounced starvation are as follows:

The first assessment shall be offered promptly, normally at the latest within 14 days of referral. If significant cardiac symptoms are reported, assessment must be made within a few days. The treatment services should be prepared to offer at least 1–2 visits a week directly at treatment start. The assessment includes the following:

- A medical assessment including a physical examination and blood tests [see national guidelines (16) for relevant tests]. An ECG should be requested in case of pronounced bradycardia. The assessment should focus on whether the patient suffers from pronounced starvation (see the manual for medical inclusion criteria).
- Detailed background history including: eating habits including developmental trajectory; physical activity; the eating disorder's impact on family and siblings; parent's efforts to help their child; basic medical history, screening for any co-morbidity.

Based on the assessment, it can be decided whether the patient is in pronounced starvation and ROCKETLAUNCH is applicable. If so, treatment begins instantly.

#### Treatment During the First Month

The treatment has a high intensity with focus on medical stabilization and weight gain from the first visit. The goal of the treatment during the first month is to gain at least 2 kg (exception made for normal or high weight AAN—where the primary goal is to achieve medical stabilization).

- The treatment always begins at the first visit. Both parents receive “parental allowance for a seriously ill child.” This is a possibility that exists in Sweden for both parents to simultaneously utilize parental leave to care for their child. This applies up to the age of 18.
- The patient is put on sick-leave from school. It is important to reconsider this decision at every visit to minimize the time the patient is excluded from their natural social context. As soon as possible a gradual return to school should be instigated.
- At the first visit, the clinician must clarify that the patient is suffering from a serious, life-threatening condition, far too severe to be managed by the young person him- or herself. Then, the clinician has to charge the parents with the full responsibility for what their child should eat and support all the meals.
- The treatment should focus on helping the parents to support each other with the difficult task of helping their child to eat. The clinician should meet with the whole family, and follow the manual. [Family therapy in case of restrictive eating disorder with pronounced starvation (first month)].
- Following the first visit, a maximum of 1 week should pass until the second visit.
- Physical assessments should be done at least weekly.
- If the patient has lost weight at the second visit, a family meal shall be scheduled within a few days. The crucial element in the family meal is to help the parents to support their ill child emotionally and help the child to eat food such as the parents believe their son or daughter needs. For guidelines on implementing the family meal, please see the manual.
- If the patient has not gained weight at the third visit, a second family meal is scheduled.
- The ROCKETLAUNCH intervention ends after 1 month and then treatment continues as usual at the local CAMHS. Most often it will be a family based treatment.

#### Family Therapy for Eating Disorders

The concept of family therapy in eating disorders has different designations at different research units. The first study was performed at the Maudsley hospital in 1987 (17) where they termed it as family therapy for anorexia nervosa (FT-AN). Family-based treatment (FBT) (18) is the most commonly used family therapy at present. It is a manualized version that has been used in most of the research on family therapy for eating disorders outside Maudsley Hospital. The Swedish manual developed for the ROCKETLAUNCH project “family therapy in case of restrictive eating disorder with pronounced starvation (first month)” is in its design very close to the FBT manual. Our unit in Lund has worked closely with the team at the Maudsley Hospital since 1988, this has affected our treatment tradition. The interventions during the first phase are very similar in both traditions and since the ROCKETLAUNCH project only covers the first month of treatment, the differences in the practical work may be negligible. There are two differences in relation to manualized FBT. The first is the family meal, the family meal session is not offered to all families. We have chosen to implement the family meal in cases where the treatment needs to be intensified when a satisfactory weight gain is not achieved. The manual for the family meal is fully in line with FBT. The second difference is when weighing the patient. This a part of a more thorough medical check-up at each family session with heart rate, blood pressure, and body temperature control. This is done by a nurse and not by the therapist.

The focus in our manual is on the practical and behavioral elements assumed to be the key components, which makes it possible to use the Swedish manual even for those who are not trained family therapists.

#### Standardized Protocols and Treatment Manuals

The above crude guidelines for the ROCKETLAUNCH project are supplemented with standardized protocols and manuals:

1. Medical inclusion criteria: a manual for medical inclusion criteria has been devised, according to the Swedish Nation Clinical Guidelines (16). The manual gives a clinically useful definition of pronounced starvation based on the results of the physical assessment. On the basis of this assessment the healthcare professional may decide whether the patient should be included in a “ROCKETLAUNCH treatment.”
2. Family therapy in case of restrictive eating disorder with pronounced starvation (first month): a Swedish manual for the family therapy based on the manuals used in the international research (18, 19). The manual describes how family therapy is carried out in the standardized care process, the guidelines outlines its core procedures.
3. Family meal: a manual in Swedish for a therapeutic family meal based on the manuals used in international research (17, 19).
4. Treatment course, physical assessment data: a standardized protocol will be used regularly during the family sessions in order to communicate clearly how the patients' physical condition changes during treatment. In this protocol data on weight, and parameters such as heart rate, blood pressure, and body temperature are collected. This is important when evaluating the outcome for AAN, as they may need weight gain but not necessarily in the same magnitude as AN.
5. Percentage of expected body weight (%EBW): when assessing underweight in children and adolescents with restrictive eating disorders, it is recommended to use the weight percentage calculated on the body mass index (BMI) value (20). In this project, we have prepared a guide for %EBW-calculation, where Swedish population norms for different ages are stated. Height stunting can affect the calculation of EBW and risks underestimating it if you only use average values. When you look at an individual child's growth curve, you can have a more valid result. In this project we choose not to look at each child's own weight curve. This protocol is a support for the physical assessment.
6. Blood pressure: a compilation of blood pressure for different ages and in relation to length, based on Swedish normal value (21) will be used. This will support the physical assessment.
7. Heart rate: a compilation of normal heart rate for different ages, based on a systematic review of current research (22) will be used.

### Training and Supervision

In order to facilitate for clinicians to work according to the manuals developed, we will carry out a training program for the healthcare professionals. The training program consists of:

- A day with lectures and seminars on the background of the project and the principles of family therapy for eating disorders.
- A training day for the local team is planned to be implemented during the first year, and will be located in Lund, Halmstad, Karlskrona, and Kalmar.
- Seminars on the guidelines in smaller groups in the workplaces, which are based on the current skill level among the staff at the various services.
- Training around family meals with those who work in the same workplace, and co-training with other units to ensure investigators that the patients are offered the same treatment.
- Supervision in the clinical work of family therapy in eating disorders, with focus on delivering the family meal, will be given continuously during the 3 years at each services, mainly by one of the authors (UW).

### Data Collection

#### Implementation Evaluation

Before the project starts, we intend to make an employee survey. The purpose of the survey is to provide each service with a basis for planning and creating favorable conditions for the implementation of ROCKETLAUNCH. In previous research (23–25), the areas that are affected in the survey proved to be important for the success of implementing new methods. In the previous implementation within Child and Adolescent Psychiatry in Sweden within the framework of the so-called “Deplyftet,” where national guidelines for the treatment of depression were implemented, an employee survey was used to identify the impeding and facilitating factors. The purpose was that each unit should have knowledge of specific factors such as:

1. Features of the innovation, the guideline itself, and the manuals in this case.
2. Features of the user of the guideline, i.e., the clinicians.
3. Characteristics of the target group, the patients.
4. Properties in the social environment and the organization.
5. Features in the system, economy, and administration
6. How similar or dissimilar is the new guideline compared to the current treatment as usual at the service level?

#### Clinical Data

##### Patients That Had Been in Treatment Before ROCKETLAUNCH

At each CAMHS data from the 10 previous patients will be gathered from the clinical records in order to compare the treatment as usual with the ROCKETLAUNCH intervention. The previous patients should all have started treatment 1 year before the start for the ROCKETLAUNCH project. The outcome variables that will be gathered are weight gain, waiting time, frequency of treatment sessions, and amount of inpatient care.

This data will include 120 patients from before the project start, and will be collected in the following manner:

- At first visit: age, waiting time from referral to the first consultation and weight and height.
- At 1 month: weight. Number of visits to reception. If the family and patient have been in day care or inpatient care, days of admission will be counted.
- At 1 year: weight and height, diagnostic status, and the days of admission will be counted.

This information about patients before the start of the project will be collected from the same time period at all services. The data from this early group will be compared with the outcome data from the ROCKETLAUNCH group.

##### The Intervention Group

**Figure 2:**
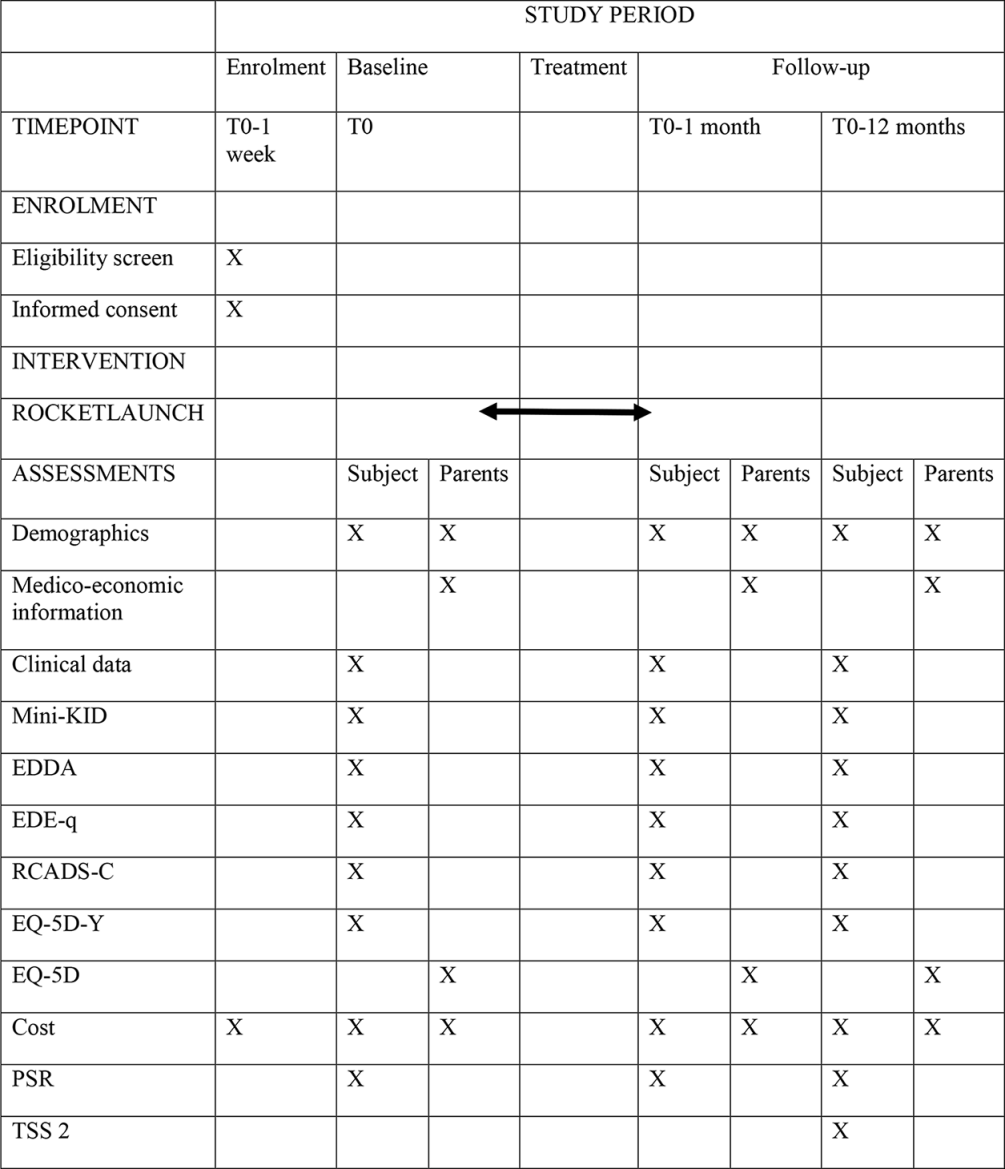
Schedule of enrolment, interventions, and assessments (SPIRIT figure).

Approximately 150 patients who will enter the project annually. A summary of the data collection is presented in **Figure 2**. Weight gain and medical data such as heart rate, blood pressure, and body temperature are regularly monitored in the treatment according to the protocol.

- At first visit: age, waiting time from notification to the first time offered and weight and height.
- At 1 month: weight. Number of visits to reception and number of visits with family meal. If the family and patient have been in day care or in-patient care, days of admission will be counted.
- At 1 year: weight and height and diagnostic status and the days of admission will be counted.

### Cost and Health Related Quality of Life

We will calculate the intervention cost, direct costs, and indirect costs. The intervention costs include training costs and costs associated with setting up and performing the intervention including the rent for the family house. Direct costs included all the cost for the healthcare sectors such as treatment, medication, foods etc. In the indirect cost, we will estimate the costs related to productivity loss of the parents while they are taking their children to the hospitals, taking care of them i.e., informal care cost and loss of income due to the intervention. The cost of informal care will be estimated by a validated questionnaire, Resource Utilization in Dementia (RUD) which is used for dementia patient (26). For the purpose of the study, the questionnaire will be modified. Overhead and administrative costs will also be calculated according to recommended methods. The patient and their accompanying parents will rated using a questionnaire about their healthcare resources utilization at baseline and also at one-month follow-up with the standard questionnaire. The questionnaire includes information regarding the primary care visits, inpatient care visits, and use of medication. For the treatment as usual group, the healthcare resources utilization will be estimated from the health registers. The registers we will use in this study are, longitudinal integration database for health insurance and labor market studies (LISA by Swedish acronym), drug or pharmaceutical register (Läkemedelsregistret), National Patient Register (Patientregistret), Cause of Death Register (Dödsorsaksregistret), Region Skåne's Health Care Utilisation Database (RSVD). The Swedish unique personal identification number will be used to identify the patients and their parents and all the registers will be linked to get the information on primary care, inpatient care, outpatient care, and use of medication. We will also use these registers to get the same information from the intervention group and their parents at 1-year follow-up.

Health related quality of life in patients and their parents will be estimated by using EQ-5D questionnaire. The investigators will use two different: one for the patient (EQ-5D-Y) (27), and one for parents (EQ-5D) (28). These two questionnaires should be completed both at the start of treatment and at the conclusion of the treatment. The questionnaires will be used to convert into a unity measures, quality adjusted life years (QALY) using the Swedish tariff.

### Instruments

The investigators will also look at psychological improvement, and evaluate the improvement in specific eating disorders psychopathology and improvement in general psychiatric psychopathology.

The course of the eating disorder is primarily intended to be followed through the national quality register “RIKSÄT,” and the additional modules that are there. The supplement to RIKSÄT is called the FEDiCS (Feeding and Eating Disorder Clinical Support System). FEDiCS is developed to be used in clinical practice. After the ROCKETLAUNCH project has ended, the CAMHS are encouraged to continue to use these instruments in the clinical assessment.

The following is included in FEDiCS:

- EDDA Standardized diagnostic interview based on Diagnostic and Statistical Manual of Mental Disorders 5 (DSM 5), which enables the interviewer to assign an eating disorder diagnosis. This instrument has recently been developed, and not yet validated. It can be used from the age of 12. (Prepared by RIKSÄT).
- MINI kid (29). A structured psychiatric diagnostic interview schedule for DSM-IV.
- EDE-Q (Eating Disorder Examination—questionnaire) (30). A self-assessment questionnaire for eating disorder symptoms. It consists of four subscale scores, relating to dietary restraint, eating concerns, concerns about weight and shape are derived from the 22 items addressing attitudinal aspects of eating-disorder psychopathology. This has been part of the national evaluation system STEPWISE for eating disorders since 2005, and the Swedish version has been studied and proven to be of good validity (31). For patients from the age of 12.
- PSR (Psychiatric Status Rating Scale) (32). Clinical assessment of the severity of the eating disorder and if the eating disorder is in remission. Rating is made by the interviewer.
- RCADS-C (Revised Children's Anxiety and Depression Scale and Subscales) (33–35). A self-rating questionnaire which is a screening for symptoms of anxiety, obsession, compulsion, and depression. For patients from the age of 12 years. RCADS consists of 47 items developed to measure DSM-IV relevant symptoms of anxiety disorders and depression in children.
- TSS-2 (Treatment Satisfaction Scale version 2) (36). A Swedish self-rating questionnaire that measures how satisfied the patient is with the treatment. For patients from the age of 12 years.

### The Therapists

The therapists will complete a questionnaire that follows the two family therapy manuals “Family therapy in case of restrictive eating disorder with pronounced starvation (first month)” and “Family meal” in order to describe the fidelity to the manual. One questionnaire for each family therapy will be used.

### Economic Evaluation

Cost-effectiveness analysis will be performed from patient, healthcare, and societal perspectives. In the societal perspective, all costs are included irrespective of who is burdened by them, while the healthcare perspective is only concerned with costs relating to the healthcare sector. The patient perspective focuses on the costs to the patient and caregiver relating to the intervention. The results will be presented in terms of incremental cost-effectiveness ratios (ICERs), which show the change in costs for an incremental benefit (37). We will perform two types of economic evaluations: 1) cost-effectiveness analysis (CEA), 2) cost utility analysis (CUA). In CEA, we will use the clinical effectiveness of the intervention such as weight gain after 1 month and at 1-year follow-up. In CUA we will use utility values using the EQ-5D questionnaires which provide the health-related quality of life as quality adjusted life years (QALY). The Swedish tariff will be used to estimate the QALY (38). The QALY for the standard care group might not be available from the registers. In this case, we will estimate the QALY for standard care for these types of patients and their caregivers from published scientific literature. The maximum willingness to pay for a QALY will be assumed to be 430,000 SEK (45,000 Euro) according to a recently published Swedish study (39).

We will perform the economic evaluation in two different time frames; short-term and long-term. In the short-term, we will calculate the cost and effects after 1 month of the intervention and in the long-term we will estimate the costs and effects with 1-year follow up data. We will analyze uncertainty by both one-way and multi-way sensitivity analysis, and also by non-parametric bootstrapping. We will calculate 95% confidence intervals for the ICERs.

### Outcome

The primary outcome measures are BMI and medical stabilization. BMI is calculated in relation to the patients expected BMI (%EBW). The change is analyzed at baseline and after 1 month and 12 months. Medical stabilization is measured as the rate of participants that have normalized heart rate and blood pressure during the first month. The secondary outcome measures are amount of days the patient needs to be hospitalized due to medical risk, change in nature, and seriousness of eating-disorder symptoms as measured with EDE-Q (Eating Disorder Examination—questionnaire) at baseline, after 1 month and after 12 months and change in emotional symptoms as measured by RCADS after 1 month and after 12 months. The ARFID group will be will analyzed separately to evaluate the effect of the intervention compared to the other patients. Other pre-specified outcome measures are the therapists' treatment fidelity. This will be evaluated as the percentage of therapists in the study following the treatment manual “Family therapy in case of restrictive eating disorder with pronounced starvation (first month).” Treatment fidelity will be measured with a questionnaire that is designed based on the treatment manual and will capture the essential treatment interventions.

### Timetable

During the four years, the clinical data and data from the FEDiCS will be collected. During the first year, the project will focus on training in the treatment method, and supervision. The investigators will start collecting data from the various units. During the second year the investigators will continue collecting data, and the supervision. During the third year, the clinical activities of the project and the supervision continue. Evaluation of the project becomes an important part in the third year. During the fourth year the investigators will compile data from the 3 years and process and begin writing reports and articles.

### Statistical Analyses

As for the primary objective, we will compare the development of %EBW between baseline after 1 month and after 12 months follow-up in the ROCKETLAUNCH group. At first, we will compare whether there was a difference in weight gain in the intervention group from baseline to 1 month. We will also compare the differences in %EBW from baseline to 12 months and 1 to 12 months. Thereafter we will compare the inter group differences, intervention group *vs*. historical control group in terms of %EBW at two different points of time 1 month and 12 months. We will use inferences statistics such as students t-test and analysis of covariance (ANCOVA) to compare the intra group and inter group differences. Furthermore, bivariate and multivariate analyses will assess the effect of the intervention while controlling for other factors such as age and gender. We plan to perform linear regression for continuous variables and logistic regression for categorical variables with 95% confidence intervals.

## Discussion

The primary objective of the project is to improve the prognosis for young newly diagnosed patients with severe restrictive eating disorders, primarily AN, and to reduce the need for in-patient psychiatric care. To achieve this, the project aims to increase skill levels so that the CAMHS may offer equal care regardless of where the patients live. According to the national quality register RIKSÄT, only 26.4% of children and young people with eating disorders in Sweden receive the evidence-based family therapy. In addition, 25% are admitted to inpatient care as the first treatment intervention. There is a need to increase the accessibility of effective treatment and to prevent unnecessary inpatient care.

By implementing key components of evidence-based family therapy during the first month of treatment, we hope to reach more patients and families than if we had tried to implement the full evidence based FBT model where the treatment runs for 20 sessions during a year. The first month of treatment is also the most crucial, when it is most important to achieve change.

## Limitations of the Study

The main limitation of the ROCKETLAUNCH project may be the challenge of involving many different CAMHS in southern Sweden. Some of the units are stable and the staff have worked together for several years, while others have clinicians with less experience. The employee survey that we plan to perform before the project starts will provide us with information that will help us provide proper training and supervision to the various units.

## Ethics Statement

The studies involving human participants were reviewed and approved by Swedish Ethical Review Authority (Dnr 2019-01852). Written informed consent to participate in this study was provided by the participants' legal guardian/next of kin.

## Author Contributions

UW is responsible for the study, and SS is responsible for health economic part.

## Funding

The project has received grants from the Kamprad Family Foundation.

## Conflict of Interest

The authors declare that the research was conducted in the absence of any commercial or financial relationships that could be construed as a potential conflict of interest.
